# A Statistical Texture Model of the Liver Based on Generalized N-Dimensional Principal Component Analysis (GND-PCA) and 3D Shape Normalization

**DOI:** 10.1155/2011/601672

**Published:** 2011-10-16

**Authors:** Xu Qiao, Yen-Wei Chen

**Affiliations:** College of Information Science and Engineering, Ritsumeikan University, 525-0054 Kusatsu, Japan

## Abstract

We present a method based on generalized N-dimensional principal component analysis (GND-PCA) and a 3D shape normalization technique for statistical texture modeling of the liver. The 3D shape normalization technique is used for normalizing liver shapes in order to remove the liver shape variability and capture pure texture variations. The GND-PCA is used to overcome overfitting problems when the training samples are too much fewer than the dimension of the data. The preliminary results of leave-one-out experiments show that the statistical texture model of the liver built by our method can represent an untrained liver volume well, even though the mode is trained by fewer samples. We also demonstrate its potential application to classification of normal and abnormal (with tumors) livers.

## 1. Introduction

In the *recent* years, digital atlases of human anatomy have become popular and important topics in medical image analysis research [1, 2]. For interpretation of images of structures and variations in the organs of the human body, it is important to have a model of the way organ volumes can be represented.

The digital atlas can be categorized as a statistical shape atlas (statistical shape model) and a statistical appearance (volume) atlas (statistical appearance (volume) model). The statistical shape model focuses on the shape information, such as feature points and volume surface [3]. It is a useful tool for study of variations in anatomic shape and has been widely used in medical image analysis, for example, medical image segmentation [4–6] and shape registration [7]. The statistical appearance model is focused on both shape and texture (voxel intensity) information. Inspired from the works of active shape models (ASMs) [3], the authors of [5, 8] proposed 3D ASMs for construction of 3D statistical models for segmentation of the left ventricle of the heart. In [9], the authors extended the work on active appearance models (AAMs) [10], and propose the use of 3D AAMs for the segmentation of cardiac MR and ultrasound images. Also, work [11] was done to build the 3D statistical deformation models (SDMs) for 3D MR brain images. Radiologists are mainly depending on the intensity variations (texture information) in livers on medical images to identify modules or tumors and make a diagnostic decision. However, there has been little research on applications of digital atlas to computer-assisted diagnosis (CAD). We have shown the potential application of statistical shape models to the classification of normal and cirrhotic livers [12]. Because many diseases will change the texture (voxel value) of the organ significantly, we need to capture not only shape variations, but also texture (voxel value) variations. Compared to statistical shape modeling, statistical texture modeling usually faces overfitting problems, and the statistical texture modeling for medical volumes is a challenging task because the dimensions of the medical volume are very high, while the training samples are much fewer than the dimensions of the data.

In our previous work, we have proposed a tensor-based subspace learning method named generalized N-dimensional principal component analysis (GND-PCA) for statistical appearance modeling of medical volumes [13]. The high-dimensional volume is treated as a 3rd-order tensor, and the optimal subspace on each mode is calculated simultaneously by minimizing of the square error between the original tensor (volume) and the reconstructed tensor (volume), based on the subspace with an iteration algorithm. As an improvement on our previous work [13], we propose a framework for capturing texture variations of the liver by using GND-PCA together with a 3D shape normalization technique (a nonrigid registration technique). The GND-PCA is used to overcome the overfitting problem, and the 3D shape normalization technique is used for normalizing liver shapes to remove the liver shape variability and capture pure texture variations. The leave-one-out experiments show that the statistical texture model of the liver built by our method can represent an untrained liver volume well, even though the model is trained by fewer samples. The preliminary results also show that the features extracted by the statistical texture model have the capability of discrimination for different types of volume data, such as normal and abnormal (with tumors).

The rest of the paper is organized as follows. In Section 2, we introduce our methodology. In Section 3, we present the experimental evaluation of our approach after introducing the datasets we used.  Section 4 concludes the paper by summarizing the main points of our contribution.

## 2. Methodology

**Figure 1:**
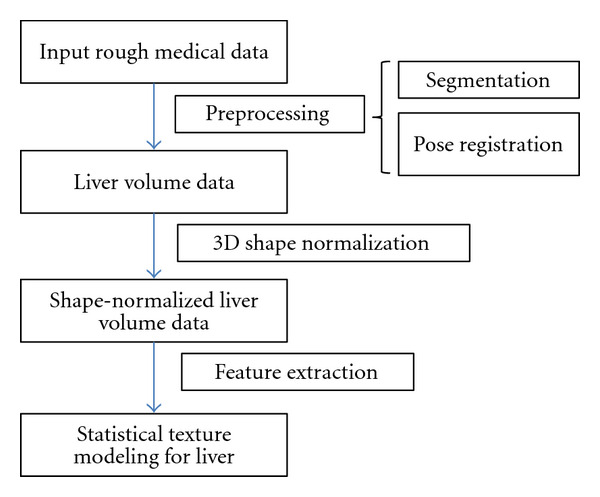
Basic scheme of statistical texture modeling.

Our proposed method for statistical texture modeling consists of two steps: (1) employing a nonrigid transformation for 3D shape normalization and (2) applying the GND-PCA method for feature extraction. The basic scheme is presented in Figure 1.

### 2.1. 3D Shape Normalization

In order to remove shape variations, we apply a nonrigid transformation based on mathematical forms for normalizing all of the datasets to the same shape. This is because the mathematical nonrigid transformations are simpler, and they can make the registration faster. Additionally, we do not need to assume the physical parameters, which are difficult to guess in practice. Hence, we adopted the mathematical nonrigid transformation in our research.

Here, we applied rigid transformation for global transformation and B-spline transformation for local transformation. The combination of global and local transformations can be represented by

(1) $$T\left(x\right)=T_{\text{Global}}\left(x\right)+T_{\text{Local}}\left(x\right),$$

where **x** = [*x*,*y*,*z*]^*T*^ is the coordinate of a 3D point.

A rigid transformation is expressed by

(2) $$T_{\text{Global}}\left(x\right)=Rx+t,$$

where **R** is the rotation matrix which can be calculated from the rotation angles ***θ*** = [*θ*_*x*_,*θ*_*y*_,*θ*_*z*_]^*T*^ around each axis. **t** is the translation vector **t** = [*t*_*x*_,*t*_*y*_,*t*_*z*_]^*T*^ along each axis. There are 6 parameters that should be estimated.

The local motion is described by cubic B-spline-based free-form deformation (FFD) modeling [14, 15]. FFD is based on locally controlled functions such as the B spline and has been applied successfully to image registration. The basic idea of FFD is to deform an object by manipulating an underlying mesh of control points. B spline transformation is defined on a regular mesh of control points with uniform spacing. Let **ρ** = [*ρ*_*x*_,*ρ*_*y*_,*ρ*_*z*_]^*T*^ be the spacing of the control points along each axis. The coordinate of a control point can be expressed by

(3) $$\varphi_{ij}={\left[\varphi_{ijk,x},\varphi_{ijk,y},\varphi_{ijk,z}\right]}^{T}={\left[i\rho_{x},j\rho_{y},k\rho_{z}\right]}^{T},$$

where *i*, *j*, *k* are the sequence number of the control points. Given the coefficients (translations) of the control points denoted as ***λ***_*ij*_ = [*λ*_*ijk*,*x*_,*λ*_*ijk*,*y*_,*λ*_*ijk*,*z*_]^*T*^, the B-spline transformation of a point **x** can be expressed by

(4) $$\begin{array}{cc} T_{\text{Local}}\left(x\right) & =\underset{ijk}{\sum }\lambda_{ijk}\beta^{\left(3\right)}\left(\frac{x-\varphi_{ijk,x}}{\rho_{x}}\right)\beta^{\left(3\right)}\left(\frac{y-\varphi_{ijk,y}}{\rho_{y}}\right)\\ & \;\times \beta^{\left(3\right)}\left(\frac{y-\varphi_{ijk,z}}{\rho_{z}}\right), \end{array}$$

where *β*^(3)^(*a*) is the third order cubic B-spline kernel. The coefficients of the control points, ***λ***_*ijk*_, are the parameters of the B-spline transformation.

The parameters of global and local transformation are optimized separately [16]. We applied software in matlab named nonrigid B-spline grid image registration toolbox [17], which is based on FFD.

### 2.2. GND-PCA Method

Modeling for medical images is an important task in medical image analysis. The principal component analysis (PCA) method [18] is an efficient method for building statistical appearance models. In the PCA-based face representation and recognition methods, the 2D face image matrices must be previously transformed into 1D image vectors column by column [19]. Such an unfolding process causes two problems; one is the huge calculation cost and another is the poor performance to be generalized.

To overcome these problems, a new technique called 2-dimensional principal component analysis (2D-PCA) [20] has been proposed, which directly computes eigenvectors of the covariance matrix of the image without matrix-to-vector conversion. It was reported that the recognition accuracy with 2D-PCA on several face databases was higher than that with conventional 1D-PCA. However, the main disadvantage of 2D-PCA is that it needs many more coefficients than that with 1D-PCA for image representation. A method called generalized 2-dimensional principal component analysis (G2D-PCA) [21] has been proposed for finding the optimal basis for both row- and column-mode subspaces.

Recently, a method called N-dimensional PCA (ND-PCA) was proposed for high-dimensional data analysis [22]. In this method, the high-dimensional data are treated as a higher-order tensor which is directly trained to obtain the bases on one mode subspace by higher-order singular value decomposition (HOSVD) [23, 24]. This method was applied to 3D scanning data. Because ND-PCA only compresses the data on one mode subspace, it also suffered from the problem that the data cannot be represented efficiently, similar to the problem of 2D-PCA.

 Inspired by the framework of generalized 2-dimensional principal component analysis [21] and N-dimensional principal component analysis [22], in our previous work, we proposed a method called generalized N-dimensional principal component analysis (GND-PCA). The high-dimensional data are treated as a series of higher-order tensors, and the optimal subspace on each mode is simultaneously calculated by minimizing the square error between the original tensor and the reconstructed tensor based on the subspace with an iteration algorithm.

**Algorithm 1**

**Figure 2:**
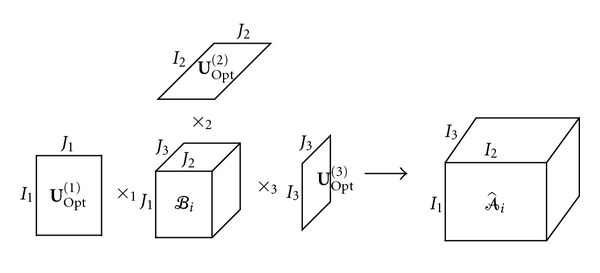
Reconstruction of a three-order tensor by the three orthogonal bases of mode subspace.

GND-PCA is formalized as follows. Given a series of the *N*-order tensors with zero means, *A*_*i*_ ∈ **R**^*I*_1_×*I*_2_×⋯×*I*_*N*_^, *i* = 1,2,…, *M*, *M* is the number of samples. We hope to get another series of low-rank (*J*_1_, *J*_2_,…, *J*_*N*_) tensors *𝒜*_i_* which accurately approximate the original tensors, where *J*_*n*_ ≤ *I*_*n*_. The new series is decomposed by the matrices *U*^(*n*)^ ∈ *R*^*I*_*n*_×*J*_*n*_^ with orthogonal columns according to Tucker's model [24], which is shown by

(5) $${𝒜}_{i}^{*}={ℬ}_{i}\times_{1}U^{\left(1\right)}\times_{2}U^{\left(2\right)}\times \cdots \times_{n}U^{\left(n\right)}\times \cdots \times_{N}U^{\left(N\right)},$$

where *ℬ*_*i*_ ∈ **R**^*J*_1_×*J*_2_×⋯×*J*_N_^ are core tensors. An illustration of reconstructing a third-order tensor by three orthogonal bases is shown in Figure 2.

The orthogonal matrices **U**^(*n*)^ can be determined by minimizing the cost function as

(6) $$C=\overset{M}{\underset{i=1}{\sum }}{||{𝒜}_{i}-{ℬ}_{i}\times_{1}U^{\left(1\right)}\times_{2}U^{\left(2\right)}\times \cdots \times_{n}U^{\left(n\right)}\times \cdots \times_{N}U^{\left(N\right)}||}^{2}.$$

Supposing that the rank of the *N* matrices **U**^(*n*)^ is known, we use an iteration algorithm to obtain the N optimal matrices, **U**_Opt_^(1)^, **U**_Opt_^(2)^,…, **U**_Opt_^(*N*)^, which are able to minimize the cost function *C*.

Here, each matrix **U**^(*n*)^ contains a set of basis vectors. An input sample can be calculated as a core tensor with the benefit of **U**^(*n*)^. This core tensor is the feature of the input sample.

Details about GND-PCA can be found in [13].

## 3. Experimental Results

### 3.1. Datasets and Preprocessing Step

**Figure 3:**
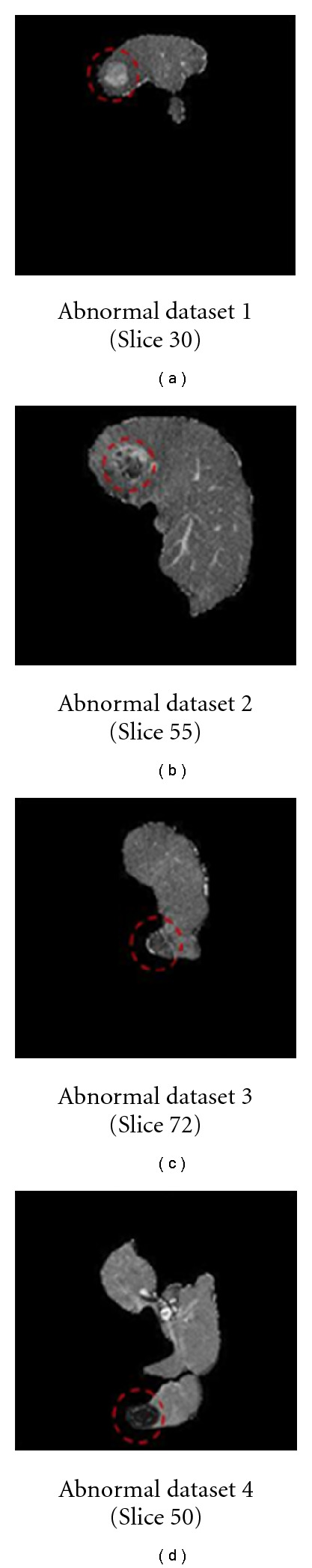
Slices of abnormal datasets.

The dataset we used to test the proposed method contains 23 abdominal CT scans collected from 23 patients, taken under similar conditions of illuminations and scanner setting. Each dataset obeys these conditions: slice thickness 2.5 mm, pitch 1.25 mm, 256 × 256 matrix, and 79 slices. This dataset contains 19 cases with no radiologic finding (noted as *normal*) and 4 cases with radiologic finding (noted as *abnormal*).  Figure 3 illustrates slices of abnormal datasets with tumors (red circles label the tumor positions).

**Figure 4:**
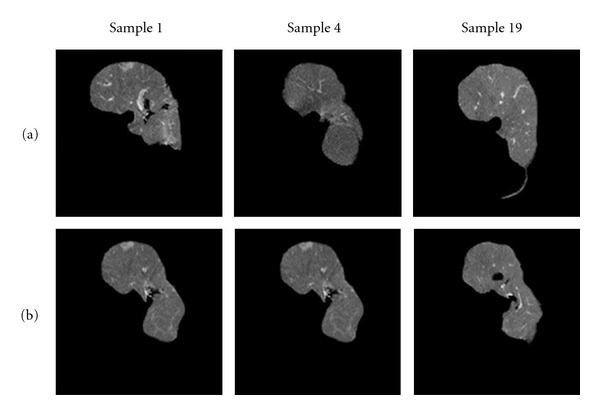
Example of datasets (some slices). (a) Original datasets. (b) 3D shape normalized datasets.

The dimension of each sample is 256 × 256 × 79. Initially livers are segmented manually from the datasets. Then we apply a rigid registration [9] for position normalization. Such pretreated datasets are noted as *original datasets*. As we mentioned in the previous section, we also apply a nonrigid registration to the dataset for both position and shape normalization to remove shape variations. The shape-normalized volumes are noted as *3D shape-normalized datasets*. Some original datasets and their 3D shape-normalized data are shown in Figure 4.

### 3.2. 3D Shape Normalization Step

**Figure 5:**
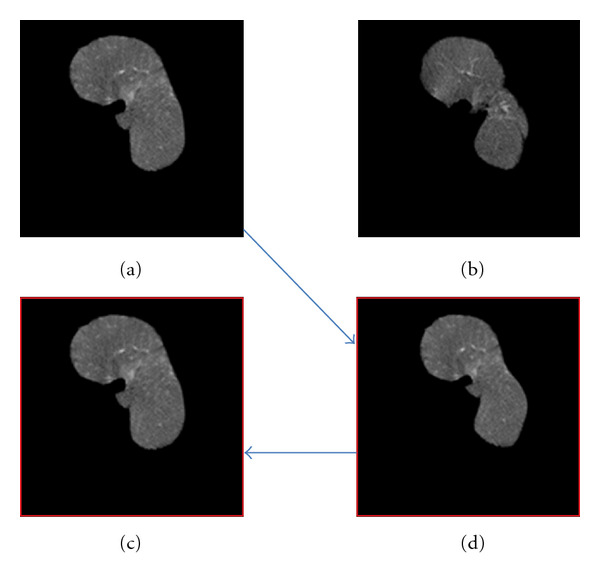
3D shape normalization processing (one slice from volume data). (a) Moved image. (b) Fixed image. (c) Shape normalized moved image. (d) Inverse of 3D shape normalization.

We show the effectiveness of shape normalization in Figure 5. Here, Figure 5(a) is one slice of moved-volume dataset, and Figure 5(b) is the corresponding slice of fixed-volume dataset. Figure 5(c) is the normalized slice of the moved volume dataset. In order to show that 3D shape-normalization processing causes little loss of texture information while interpolate the pixel values for shape deformation, we apply 3D shape normalization to the normalized moved-volume dataset again to transform it back to the original shape. Comparison of the inverse slice (Figure 5(d)) with the original slice (Figure 5(a)) shows that 3D shape normalization processing keeps almost all texture information. Thus, it is reasonable to apply 3D shape normalization as a preprocessing step to remove shape variations. 

In our experiment, we chose the B-spline grid dimensions as (26 26 8), and we randomly chose one dataset as the fixed volume and normalized the other dataset to the same shape.

### 3.3. Modeling for Generalization

**Figure 6:**
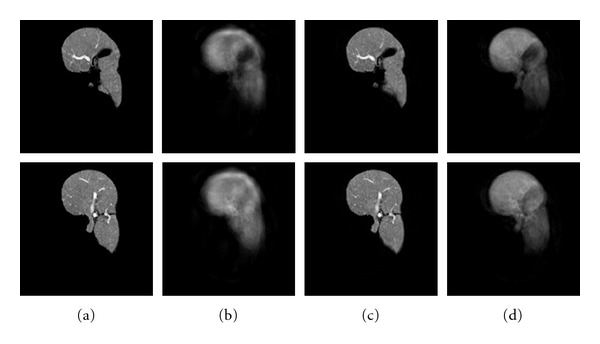
Reconstructed results for two slices of one test sample without shape normalization. (a) Original images. (b) Images reconstructed with 10 × 10 × 4  basis by GND-PCA. (c) Images reconstructed with 100 × 100 × 40  basis by GND-PCA. (d) Images reconstructed by PCA (eigenface method); all 15 available bases are used in the reconstruction.

**Figure 7:**
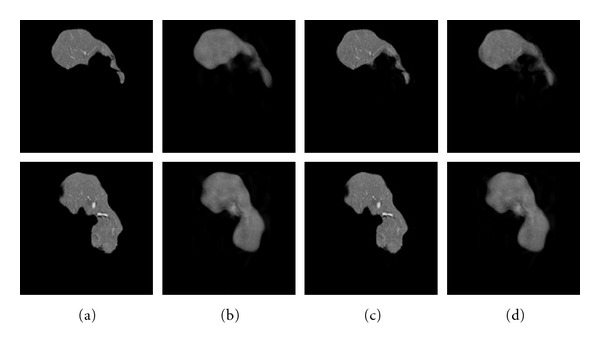
Reconstructed results for two slices of one test sample after shape normalization. (a) 3D shape-normalized images. (b) Images reconstructed with 10 × 10 × 4 basis by GND-PCA. (c) Images reconstructed with 100 × 100 × 40 basis by GND-PCA. (d) Images reconstructed by PCA (eigenface method); all 15 available bases are used in the reconstruction.

**Figure 8:**
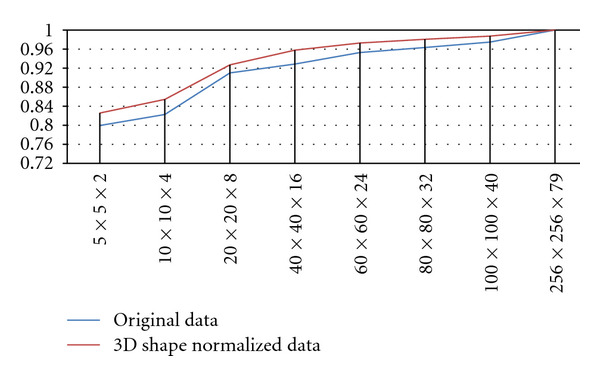
Normalized correlations while basis increasing.

The proposed GND-PCA is applied to both original and shape-normalized datasets. The leave-one-out experiment is done to test the generalization ability of GND-PCA. As a small number of abnormal datasets of the liver, we randomly used 15 datasets to learn the optimal subspaces, and of the others the one left untrained was used as an input. Typical results are shown in Figures 6 and 7. The test volume was reconstructed from 10 × 10 × 4 and 100 × 100 × 40 mode-subspace bases by GND-PCA, respectively.  Figure 8 illustrated that the reconstructed images were improved by an increase in the subspace basis. In spite of having very few samples, we still could obtain an almost perfect reconstruction with 100 × 100 × 40 basis. In order to make a comparison, we also show the reconstructed results by the conversional PCA (eigenface) method in Figures 6(d) and 7(d), which show that the quality of the reconstructed results are not satisfied even though the entire 15 available bases are used for reconstruction because of overfitting.

The normalized correlations between the original volume and the reconstructed volume are shown in Figure 8. Compared with in the case of the original dataset, the datasets can be represented by a small number of bases in the case of shape-normalized dataset because the subspace contains only texture variations.

### 3.4. Modeling for Discrimination

Next, we introduce a simple experiment to show that the features extracted by our methods have the capability for discrimination. We used only 15 normal datasets for training and left the other 8 datasets for testing. The testing samples included 4 normal datasets and 4 abnormal datasets. After we obtained the optimal subspace by the GND-PCA method, each sample was represented by a core tensor. The core tensor is a feature of the sample and is noted as *ℬ*_*i*_. We also calculate the mean feature of all of the training datasets and noted it as *ℬ*_Center_. Here, the dimension of the core tensor is 100 × 100 × 40.

The Euclidean distance (ED) is applied to the calculation of the distance between *ℬ*_*i*_ and *ℬ*_Center_.  Table 1 shows the ED for all the testing samples. Compared with those in the original datasets, the distances decreased in the shape-normalized dataset's experiments. We demonstrated that shape variations are removed by 3D shape normalization.

**Figure 9:**
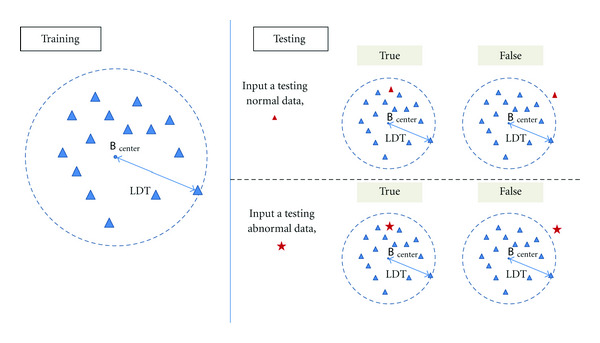
The rule for judging a test sample as normal or abnormal (2D case as example).

Next, we showed how to identify the normal datasets and abnormal datasets. The features captured by our method are tensor formed; they can be flatten as high-dimensional vectors. In order to separate the features into two classes: normal and abnormal, we need to find a high-dimensional hyperplane. It is difficult to describe the hyperplane in high-dimensional space; we use Figure 9 as a 2D case to show how to find a hyperplane. Compared with the normal datasets, abnormal datasets have some significant parts in texture. If we do not consider the effect of shape, the significant parts caused a higher value of ED for abnormal datasets because we only used normal samples for training. We used the largest ED of the training sample (LDT), which is also shown in Table 1, as a boundary of normal and abnormal for classification.  Table 2 gives the classified results for two kinds of dataset experiments. It demonstrates that the features extracted by our method have better performance for discriminations between the normal and abnormal classes.

## 4. Conclusion

In this paper, we developed a statistical texture modeling method for medical volumetric images based on 3D shape normalization and GND-PCA. We first propose to use a 3D shape normalization technique to normalize all volume datasets to the same shape to obtain the 3D shape-normalized datasets, which can be considered to contain only the texture variations. Then we trained them to construct the statistical model only for texture by GND-PCA method for application to liver volumes. Reconstruction results show a good performance on generalization by using our proposed method. We also designed a simple experiment to identify different types of data with corresponding features, such as normal and abnormal, which proved that the proposed model can be used for computer-assisted diagnostics of liver disease. In the future, we will test our method with more datasets for classification and use our method in practical applications.

## Figures and Tables

**Table 1:** Euclidean distances of features.

	Original data		3D shape-normaized data	
	ED	ED-LDT	ED	ED-LDT
LDT	26984	0	13817	0

Normal dataSet_1	29101	2117.5	13339	−477.55
Normal dataSet_2	20394	−6589.4	8474.4	−5342.3
Normal dataSet_3	16811	−10173	11872	−1944.7
Normal dataSet_4	21584	−5399.6	9432.6	−4384

Abnormal dataSet_1	25896	−1087.8	18400	4583.8
Abnormal dataSet_2	29633	2649.2	17314	3497.1
Abnormal dataSet_3	23303	−3680.3	16502	2685.6
Abnormal dataSet_4	30405	3421.6	19241	5424

**Table 2:** Classification result.

	Class	Test sample number	Correct classified number	Accuracy
Original data experiment	Normal	4	3	75%
	Abnormal	4	2	50%

3D shape normalized data experiment	Normal	4	4	100%
	Abnormal	4	4	100%
